# Making Nd^3+^ a Sensitive Luminescent Thermometer for Physiological Temperatures—An Account of Pitfalls in Boltzmann Thermometry

**DOI:** 10.3390/nano10030543

**Published:** 2020-03-18

**Authors:** Markus Suta, Željka Antić, Vesna Ðorđević, Sanja Kuzman, Miroslav D. Dramićanin, Andries Meijerink

**Affiliations:** 1 Condensed Matter and Interfaces, Debye Institute for Nanomaterials Science, Department of Chemistry, Utrecht University, Princetonplein 1, 3584 CC Utrecht, The Netherlands; a.meijerink@uu.nl; 2 Vinča Institute of Nuclear Sciences, University of Belgrade, 11001 Belgrade, Serbia; zeljkaa@gmail.com (Ž.A.); vesipka@vin.bg.ac.rs (V.Ð.); sanjaculubrk@gmail.com (S.K.); dramican@gmail.com (M.D.D.)

**Keywords:** Nd^3+^, luminescence thermometry, in vivo imaging, Boltzmann equilibrium, time-resolved spectroscopy

## Abstract

Ratiometric luminescence thermometry employing luminescence within the biological transparency windows provides high potential for biothermal imaging. Nd^3+^ is a promising candidate for that purpose due to its intense radiative transitions within biological windows (BWs) I and II and the simultaneous efficient excitability within BW I. This makes Nd^3+^ almost unique among all lanthanides. Typically, emission from the two ^4^*F*_3/2_ crystal field levels is used for thermometry but the small ~100 cm^−1^ energy separation limits the sensitivity. A higher sensitivity for physiological temperatures is possible using the luminescence intensity ratio (LIR) of the emissive transitions from the ^4^*F*_5/2_ and ^4^*F*_3/2_ excited spin-orbit levels. Herein, we demonstrate and discuss various pitfalls that can occur in Boltzmann thermometry if this particular LIR is used for physiological temperature sensing. Both microcrystalline, dilute (0.1%) Nd^3+^-doped LaPO_4_ and LaPO_4_: *x*% Nd^3+^ (*x* = 2, 5, 10, 25, 100) nanocrystals serve as an illustrative example. Besides structural and optical characterization of those luminescent thermometers, the impact and consequences of the Nd^3+^ concentration on their luminescence and performance as Boltzmann-based thermometers are analyzed. For low Nd^3+^ concentrations, Boltzmann equilibrium starts just around 300 K. At higher Nd^3+^ concentrations, cross-relaxation processes enhance the decay rates of the ^4^*F*_3/2_ and ^4^*F*_5/2_ levels making the decay faster than the equilibration rates between the levels. It is shown that the onset of the useful temperature sensing range shifts to higher temperatures, even above ~ 450 K for Nd concentrations over 5%. A microscopic explanation for pitfalls in Boltzmann thermometry with Nd^3+^ is finally given and guidelines for the usability of this lanthanide ion in the field of physiological temperature sensing are elaborated. Insight in competition between thermal coupling through non-radiative transitions and population decay through cross-relaxation of the ^4^*F*_5/2_ and ^4^*F*_3/2_ spin-orbit levels of Nd^3+^ makes it possible to tailor the thermometric performance of Nd^3+^ to enable physiological temperature sensing.

## 1. Introduction

Temperature is an important control parameter that governs, e.g., the rate of chemical reactions, but also the optimum working efficiency of electronic devices or dynamics and viability of biological systems. As such, non-invasive, sensitive and remote temperature measurement techniques with the capability to spatially resolve temperature variations down to the micrometer range are becoming increasingly relevant [1,2,3,4,5,6,7]. Physiological temperature sensing is especially demanding in that regard as it even requires to accurately distinguish temperature fluctuations below 1 K. Luminescence nanothermometry is an appealing and rapidly emerging technique that meets those requirements and constantly improves [8,9,10,11,12,13,14]. Among the various temperature-dependent optical parameters that can be used for temperature calibration, the luminescence intensity ratio (LIR) of two emissive transitions from thermally coupled excited states has emerged as an especially robust and yet easily measurable representative to extract information about the local temperature of a medium in contact with luminescent nanocrystals. Also, other methodological approaches such as lifetime thermometry [15,16,17,18,19,20,21], or the concept of excited state absorption (ESA) [22,23,24] have been developed for that purpose while organic framework-based thermometers increasingly attract attention [25,26].

Besides the requirement for a low temperature uncertainty (<±1 K) over a narrow temperature range (30 °C–60 °C), luminescence nanothermometry under physiological conditions has additional restrictions. Absorbance, light scattering or even autofluorescence of locally surrounding tissue pose challenges that have to be overcome to guarantee a reliable detection of the LIR outside the biological system of interest [27,28,29]. Thus, near infrared (NIR) luminescence within the biological transparency windows (BW I: 650–950 nm; BW II: 1000–1350 nm; BW III: 1550–1850 nm) is highly desirable for minimized attenuation of the emitted light to guarantee high temperature precision. While quantum dots rely on a sensitive thermal quenching behavior of their NIR luminescence within physiological temperature regimes and have successfully been employed in various biomedical applications [30,31,32,33,34,35,36], their usage in thermometry still requires careful calibration [37].

As an alternative, the lanthanide ion Nd^3+^(4*f*^3^) has become a promising candidate for in vivo nanothermometry [30,38,39,40,41,42,43]. This is related to the fact that its most intense radiative transitions all lie within BW I and BW II. Moreover, the large energy gap between the most dominantly emissive ^4^*F*_3/2_ spin-orbit level and the next lower ^4^*I*_15/2_ level typically allows for rather high brightness of Nd^3+^-activated luminescent nanocrystals. Finally, Nd^3+^ most efficiently absorbs within the first biological window. Altogether, those optical properties make Nd^3+^ almost unique among all lanthanides for NIR luminescence thermometry. Since the ^4^*F*_3/2_ spin-orbit level of Nd^3+^ splits into exactly two energetically well isolated Kramers’ doublets (*R*_1_ and *R*_2_) in any lower symmetric crystal field with an energy gap of around 100 cm^−1^, the vast majority of studies aiming at single ion in vivo nanothermometry with Nd^3+^ addressed the LIR stemming from emission from those two crystal field states [38,40,41,44,45]. Among the various possibilities of synthetically well accessible and size-controllable nanocrystals such as fluorides [46,47,48], nanosized garnets have become especially interesting in that sense due to the well resolved and intense ^4^*F*_3/2_(*R*_1,2_) → ^4^*I*_9/2_(*Z*_5_) emissive transitions at 935 nm and 945 nm. This allowed researchers to tune the relative thermal sensitivity *S*_r_ of this particular LIR towards the theoretically expected maximum value of around 0.15% K^−1^ at 37 °C according to the given energy gap [49,50]. The value obtained with that concept of Nd^3+^-based nanothermometry could only be exceeded in lattices with higher crystal field splitting, for example LiLuF_4_ nanocrystals, with a value of around 0.5% K^−1^ at 37 °C [51].

Usage of the two Kramers’ doublets for in vivo nanothermometry is, however, connected to some inherent problems. Besides the challenges in size and morphology control of nanosized garnets [52] compared to, e.g., well-established synthesis routes to fluoride nanocrystals [46,47,48], the requirement for high spectral resolution and unambiguous assignment of the two closely lying emissive transitions to be attained under real in vivo conditions such as light scattering and absorption of surrounding tissue, dynamic flow, or movement of biological entities, is challenging. Even more severely, the theoretically achievable maximum relative sensitivity is well below 1% K^−1^ and does not allow to achieve the required temperature measurement precision below 1 K even with use of laser excitation sources and sensitive light detection systems. Finally, the small energy gap between the two Kramers’ doublets inherently diminishes the absolute thermal response of the LIR because the two states have similar population densities at room temperature, as has been shown explicitly by thermodynamic modelling of a generalized excited two-level system to be reported in detail elsewhere [53].

One way to overcome this problem and achieve an order of magnitude higher relative sensitivities has already been demonstrated by a phonon-assisted energy transfer between Nd^3+^ and Yb^3+^ [30,54,55] or recently, also Nd^3+^ and Er^3+^ [43]. However, the underlying temperature calibration model for the temperature-dependent LIR based on energy transfer is far from trivial and, strictly stated, Boltzmann’s law does not apply for those situations [53]. In addition, the NIR emission of Yb^3+^ lies in between the two transparency windows BW I and II and strongly overlaps with the ^4^*F*_3/2_ → ^4^*I*_11/2_-related emission of Nd^3+^ thus introducing additional error sources for accurate ratiometric luminescence thermometry.

A promising alternative that would allow for calibration with Boltzmann’s law and does not require the introduction of additional dopants is usage of the two spin-orbit levels ^4^*F*_5/2_ and ^4^*F*_3/2_ of Nd^3+^ instead. They have an effective energy separation of roughly 1000 cm^−1^ and emission from those two levels can be easily spectrally resolved. In addition, the higher energy gap permits an order of magnitude higher relative sensitivity (*S*_r_ > 1% K^−1^) at physiological temperatures, which meets the requirements for in vivo nanothermometry. Besides an original work by Haro-González et al. in a Nd^3+^-doped Sr-Ba-based niobate glass demonstrating a proof of principle [56], a more elaborate analysis of thermometry with that energy gap has been recently reported by Kolesnikov et al. in Y_2_O_3_:Nd^3+^ nanocrystals [57]. However, thermal coupling between the ^4^*F*_5/2_ and ^4^*F*_3/2_ spin-orbit levels is an issue because of the relatively large gap of around 1000 cm^−1^. The non-radiative transition rates between the two spin-orbit levels have to be faster than decay rates of these levels within the relevant temperature regime since otherwise Boltzmann-based equilibrium is not sustained [58]. In addition, higher doping concentrations to help increase the absorption efficiency of Nd^3+^ may also induce faster decay as a result of energy migration and cross-relaxation effects, preventing Boltzmann equilibrium. Thus, dependent on the electronic structure of a lanthanide ion, the effect of energy migration or cross relaxation at higher doping concentrations on the Boltzmann equilibrium between two excited levels of interest has to be investigated in detail.

Here we present and discuss the ^4^*F*_5/2_ and ^4^*F*_3/2_ based alternative thermometric sensing by Nd^3+^ and evaluate its applicability for in vivo nanothermometry using LaPO_4_:Nd^3+^ nanocrystals as an illustrative example. The choice of the host material arises from the fact that phosphates have a large maximum phonon energy of around 1050 cm^−1^ that allows for resonant thermalization between the ^4^*F*_5/2_ and ^4^*F*_3/2_ levels [59]. Moreover, phosphate nanocrystals may also be at least theoretically expected to show biocompatibility given its presence to a large fraction in the body of mammals. Besides a structural and optical characterization, especially the impact of both temperature and Nd^3+^ concentration on the thermometric performance of those nanocrystals will be analyzed. We will carefully compare the found results to the luminescence decay kinetics of the Nd^3+^ ions and compare predictions with experimental results. Our general purpose is to draw attention to some potential pitfalls in conventional Boltzmann thermometers, discuss their origin and provide solutions.

## 2. Materials and Methods

### 2.1. Synthesis

La_1−*x*_Nd*_x_*PO_4_ nanocrystals (*x* = 0.02, 0.05, 0.10, 0.25, 1.00) were synthesized with a common hydrothermal co-precipitation approach. For that, stoichiometric amounts of La(NO_3_)_3_ · 6 H_2_O (Alfa Aesar, 99.99%, Karlsruhe, Germany) and Nd(NO_3_)_3_ · 6 H_2_O (Alfa Aesar, 99.9%, Karlsruhe, Germany) were dissolved in deionized water. A small volume of 1 M NaOH (Chemos, 99%, Altdorf, Germany) solution was added under stirring of the solution to attain an alkaline medium. Afterwards, an aqueous solution containing a stoichiometric amount of (NH_4_)_2_HPO_4_ (Alfa Aesar, 98%, Karlsruhe, Germany) was added to allow for precipitation of the rare earth phosphates. The resulting solution was transferred to a Teflon-lined stainless-steel autoclave and kept at 140 °C for 30 h to induce crystallization of the nanoseeds of the rare earth phosphates. After natural cooling to room temperature, the colorless precipitate was collected, washed with deionized H_2_O and ethanol (EtOH) several times and finally dried at 40 °C for 22 h.

Microcrystalline, dilute La_0.999_Nd_0.001_PO_4_ powder was synthesized for control experiments and to elucidate concentration effects. The synthesis was similarly performed by means of a co-precipitation approach from dissolved La(NO_3_)_3_ · 6 H_2_O (Sigma Aldrich, 99.99%, Schnelldorf, Germany) and Nd(NO_3_)_3_ · 6 H_2_O (Sigma Aldrich, 99.9%, Schnelldorf, Germany) in alkaline aqueous solution and precipitation with a saturated (NH_4_)_2_HPO_4_ (Sigma Aldrich, 99%, Schnelldorf, Germany) solution. The colorless precipitated phosphate was filtered off, washed with deionized H_2_O three times and carefully dried at 150 °C on air for 3 h. After grinding of the dried residue to a fine powder, it was finally annealed at 1000 °C in air for 3 days.

### 2.2. Structural and Morphological Characterization

The La_1−*x*_Nd*_x_*PO_4_ (*x* = 0.02, 0.05, 0.10, 0.25, 1.00) nanocrystalline powders were characterized by means of X-ray powder diffraction (XRPD) and transmission electron microscopy (TEM). XRPD patterns were measured on a SmartLab (Rigaku, Tokyo, Japan) instrument using Cu *K_α_* radiation (*λ* = 1.54056 Å). The average crystallite size and internal strain of the nanocrystals were determined by a PDXL2 built-in package software using the known crystal structures of LaPO_4_ and NdPO_4_ as a structural input. The XRPD pattern of La_0.999_Nd_0.001_PO_4_ was measured on a Philips PW391 X-ray diffractometer (Eindhoven, The Netherlands) with Cu *K_α_* radiation (*λ* = 1.54056 Å) in reflection mode with an Al sample holder.

TEM images were acquired on a Thermo Fisher Scientific (formerly Philips, Eindhoven, The Netherlands) FEI Tecnai 12 microscope with an electron acceleration voltage of *U* = 100 kV. Samples were prepared by scooping a Cu TEM grid with carbon-coated polymer support film into the nanopowders.

### 2.3. Diffuse Powder Reflectance Spectroscopy

Diffuse reflectance spectra were acquired on a Shimadzu (Kyoto, Japan) UV-Visible UV-2600 spectrophotometer equipped with an integrated sphere (ISR-2600 Plus). All diffuse reflectance spectra were corrected for background using a BaSO_4_ standard in the regarded spectral range (200–1200 nm).

### 2.4. (Time-Resolved) Luminescence Spectroscopy and Thermometry

Luminescence spectra were measured on an Edinburgh FLS920 spectrofluorometer (Livingston, UK) equipped with 0.25 m single grating monochromators and detected with a Hamamatsu R5509-72 NIR photomultiplier tube (PMT, Hamamatsu, Shizuoka, Japan) that was cooled with liquid N_2_. All emission spectra were acquired by excitation with continuous wave (CW) power-tunable (power limit: *P*_max_ = 800 mW, Livingston, UK) lasers operating at *λ*_ex_ = 690 nm. A high-resolution emission spectrum to evaluate the impact of non-radiative relaxation from the ^4^*F*_5/2_ to the ^4^*F*_3/2_ level was measured upon excitation with a CW power-tunable (power limit: *P*_max_ = 2 W, Livingston, UK) laser with *λ*_ex_ = 808 nm. The incident laser power in each experiment was set to a sufficiently low value in order to avoid laser-induced heating with yet high signal-to-noise ratio. Photoluminescence excitation spectra were acquired upon monitoring the most intense emission of Nd^3+^ at λ_em_ = 1057 nm and a 450 W Xe lamp as external excitation source. Luminescence decay curves in the microsecond domain were obtained by external excitation with a pulsed wavelength-tunable Opotek (Carlsbad, CA, USA) Opolette 355 LD optical parametric oscillator (OPO) with a repetition rate of 20 Hz and temporal pulse width of around 6 ns. The time-resolved signal was detected with a multichannel scaler (MCS) connected to the NIR PMT. Temperature-dependent emission spectra above room temperature were measured in a Linkam (Surrey, UK) THMS600 Microscope Stage (±0.1 °C temperature stability) that could be placed in the spectrometer.

## 3. Results and Discussion

### 3.1. Structural and Morphological Characterization of the Nd^3+^-Activated LaPO_4_ Nanocrystals

Both LaPO_4_ and NdPO_4_ crystallize in a monazite structure type with monoclinic crystal system and space group *P*2_1_/*n* (no. 14) [60,61]. Only one crystallographically independent La^3+^ or Nd^3+^ site on the Wyckoff position 4*e* are present in both unit cells, respectively. Given the only slightly smaller ionic radius of Nd^3+^ (1.163 Å for nine-fold coordination) compared to that of La^3+^ (1.216 Å for nine-fold coordination) [62], a full range of solubility of NdPO_4_ within the LaPO_4_ host is expected. Synthesis of the solid solutions La_1−*x*_Nd*_x_*PO_4_ by a co-precipitation approach instead conventional heterogeneous mixing and subsequent thermal annealing permits a more random distribution of the Nd^3+^ activators substituting for the La^3+^ ions, as has also been recently independently established by solid state magic angle spinning nuclear magnetic resonance (MAS-NMR) experiments on the ^31^P nuclei in lanthanide-activated LaPO_4_, whose resonances sensitively react on paramagnetic impurities in their close environment [63,64,65].

**Figure 1:**
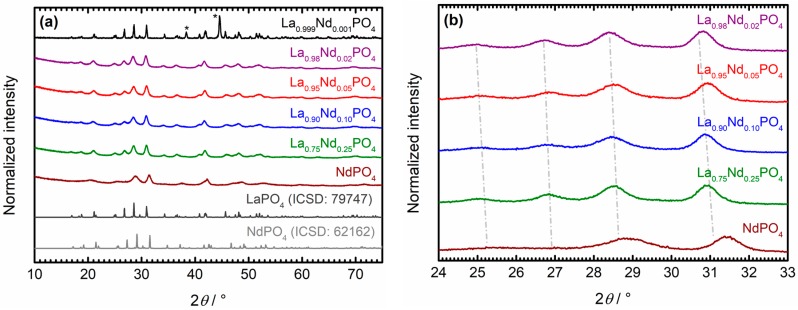
(**a**) X-ray powder diffraction (XRPD) patterns of synthesized microcrystalline La_0.999_Nd_0.001_PO_4_ and La_1−*x*_Nd*_x_*PO_4_ nanocrystals in comparison to the diffraction patterns of LaPO_4_ (ICSD: 79747) and NdPO_4_ (ICSD: 62162) as simulated from single crystal diffraction data [60,61]. The reflections marked with asterisks are due to the employed Al sample holder. The diffraction patterns were stacked for better comparison. (**b**) Enlarged view on the XRPD patterns of the La_1−*x*_Nd*_x_*PO_4_ nanocrystals over a selected 2*θ* range (24°–33°) to emphasize the gradual shift of reflections towards higher angles with increasing *x*.

Figure 1a depicts the XRPD patterns of the Nd^3+^-activated LaPO_4_ nanocrystals as prepared by the hydrothermal co-precipitation approach. The broad background in the low 2*θ* regime as well as the low intensities and large widths of most Bragg reflections already indicate a small average crystallite size of the particles. Rietveld refinement of the X-ray diffraction patterns employing the monazite structure type as structural input [60,61], affords estimated average crystallite sizes between 6 nm and 10 nm (see Table 1). The small *R* factors below 10% and the goodness of fit (G.o.f.) parameter close to 1 indicate a very good agreement between the theoretically expected powder diffraction pattern according to the structural input of the monazite-type phases and the experimentally measured diffraction patterns (see Table 1). Moreover, the resulting strain percentage within the nanocrystals is close to 0%, which reflects the miscibility of the two constituents LaPO_4_ and NdPO_4_ in the solid solution.

Additional evidence for the homogeneous distribution of the Nd^3+^ ions substituting for the La^3+^ sites in LaPO_4_ is given by observation of a gradual shift of the Bragg reflections towards higher values of 2*θ* with increasing Nd^3+^ content in the nanocrystals (see Figure 1b). The microcrystalline sample was separated from that analysis since the well-defined reflections in that particular case merge together upon particle-size induced broadening of the Bragg reflections in the nanocrystals.

This effect leads to artificial shifts to yet lower Bragg angles despite an increase of the Nd^3+^ content from *x* = 0.001 to *x* = 0.02. Accordingly, the resulting cell volume as obtained from the Rietveld refinement also gradually decreases with higher Nd^3+^ content (see Table 1).

**Figure 2:**
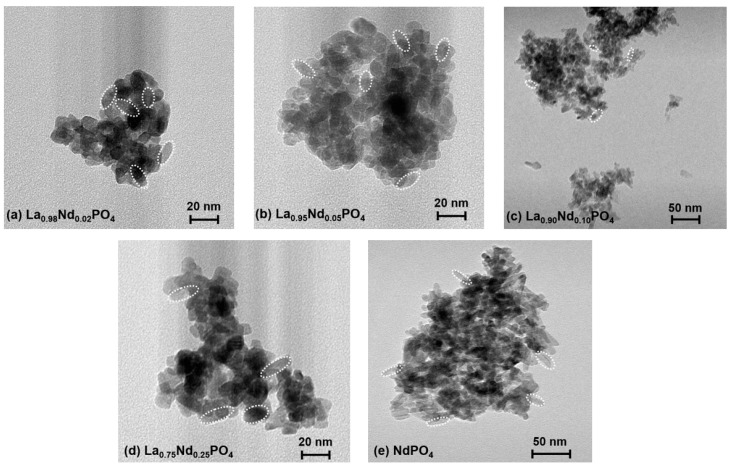
Transmission electron microscopy (TEM) images of the synthesized La_1−*x*_Nd*_x_*PO_4_ nanocrystals: (**a**) *x* = 0.02, (**b**) *x* = 0.05, (**c**) *x* = 0.10, (**d**) *x* = 0.25 and (**e**) *x* = 1.00. Scale bars indicate either 20 nm or 50 nm, respectively. Selected single nanorods are marked by white dashed ellipses.

The TEM images (see Figure 2) of the La_1−*x*_Nd*_x_*PO_4_ nanocrystals confirm the estimated crystallite sizes according to the XRPD patterns and diameters in the range of 10 nm to 15 nm are found. The nanocrystals have an anisotropic rod-like shape, which is also expected given the monoclinic crystal system. An average aspect ratio of around length/width = 1.5 is found for all nanocrystals. Moreover, the TEM images reveal a strong aggregation tendency of the nanocrystals in the powder, which was also found previously in higher condensed La-based phosphates such as the tetraphosphates ALa_1−*y*_Nd*_y_*(PO_3_)_4_ (A = Li – Rb; 0 ≤ *y* ≤ 1) [66].

### 3.2. Diffuse Reflectance and Optical Absorption

**Figure 3:**
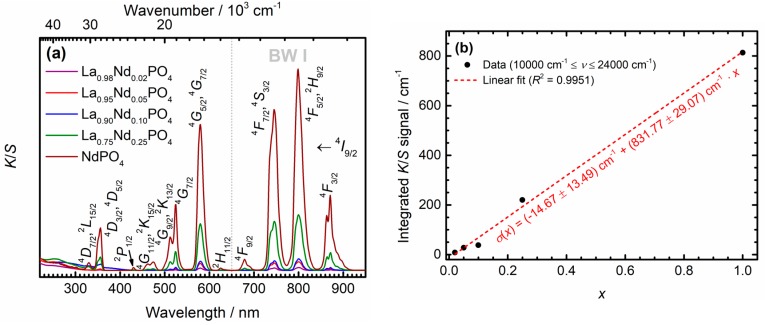
(**a**) Kubelka-Munk graphs of the La_1−*x*_Nd*_x_*PO_4_ (*x* = 0.02, 0.05, 0.10, 0.25, 1.00) nanocrystals in powder form as obtained from the respective diffuse reflectance spectra (see Equation (1)). The respective assignment of the absorption transitions and the first biological window (BW I) are indicated. (**b**) Correlation between the Nd^3+^ content *x* and the integrated absorption signal when evaluated in wavenumber scales. The red dashed line indicates a least-squares linear fit to the data. The intercept does not differ from zero at the statistical significance level of *α* = 0.05.

Diffuse reflectance spectra can serve as an additional characterization tool for the successful incorporation of Nd^3+^ ions into the synthesized Nd^3+^-activated LaPO_4_ nanocrystals. The very low Nd^3+^ concentration in microcrystalline La_0.999_Nd_0.0001_PO_4_ did not give rise to a measurable reflectance signal and was thus excluded. A measure for the absorption coefficient of the respective powdered samples is accessible by the Kubelka-Munk function *K*/*S* under the assumption of a constant scattering part of the powders:

(1) $$\frac{K}{S}=f\left(R_{\infty }\right)=\frac{{\left({1\;-\;R}_{\infty }\right)}^{2}}{{2R}_{\infty }},$$

where $R_{\infty }$ denotes the diffuse reflectance in the limit of a much higher scale of layer thickness of the powder compared to the average crystallite size, which is clearly fulfilled under the employed measurement conditions. The Kubelka-Munk spectra are depicted in Figure 3a. As expected, the narrow 4*f*^3^(^4^*I*_9/2_) → 4*f*^3^(^2*S*+1^*L_J_*) transitions of Nd^3+^ show an increasing absorption strength with increasing Nd^3+^ content of the nanocrystals. Indeed, a linear correlation between the integrated Kubelka-Munk signal—if accordingly translated to an energy scale—and nominal Nd^3+^ concentration *x* is present over the whole regime between *x* = 0 and *x* = 1, in agreement with expectations from electromagnetic dispersion theory (see Figure 3b) [67]. On a statistical significance level of *α* = 0.05, the intercept of the linear calibration line does not differ from zero, as verified by a conventional *t* test. Altogether, the diffuse reflectance spectra confirm a homogeneous miscibility between the LaPO_4_ and NdPO_4_ phases within the nanocrystals.

In the context of suitability for in vivo nanothermometry, the Kubelka-Munk spectra (see Figure 3a) also reveal that the strongest absorption transitions of Nd^3+^ within the discussed nanocrystals are located in the first biological window between 650 nm and 950 nm. This demonstrates the general suitability of Nd^3+^ as a potent absorber for in vivo applications since its absorption remains negligibly affected by light attenuation due to surrounding tissue. The linear correlation of the integrated absorption strength with concentration in the La_1−*x*_Nd*_x_*PO_4_ nanocrystals does in principle allow for a simple strategy to improve the absorption strength in the Nd^3+^-activated nanocrystals using higher Nd^3+^ contents. As will be discussed below, however, this can induce both energy migration and cross-relaxation effects that limit the temperature window and thermometric performance of the nanocrystals.

### 3.3. Photoluminescence Properties and Luminescence Decay Dynamics—Predictions on Consequences for Thermometry with Nd^3+^

**Figure 4:**
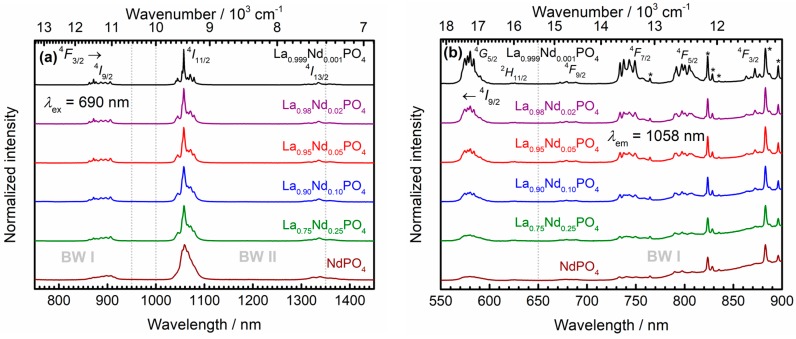
(**a**) Photoluminescence spectra of the powdered La_1−*x*_Nd*_x_*PO_4_ nanocrystals acquired upon laser excitation with 690 nm at room temperature. The respective transitions and biological windows are indicated. The microcrystalline La_0.999_Nd_0.001_PO_4_ control sample was also added. (**b**) Photoluminescence excitation spectra of the powdered La_1−*x*_Nd*_x_*PO_4_ nanocrystals acquired upon monitoring the ^4^*F*_3/2_ → ^4^*I*_11/2_ radiative transition at 1057 nm at room temperature. The respective transitions and biological window are indicated. The microcrystalline La_0.999_Nd_0.001_PO_4_ control sample was also added. Peaks marked with an asterisk stem from the employed Xe lamp and could not be removed even by usage of a 1020 nm long pass filter. All spectra were stacked for the sake of clarity.

Figure 4a depicts the photoluminescence emission spectra of the La_1−*x*_Nd*_x_*PO_4_ nanocrystals at room temperature upon CW laser excitation at 690 nm into the ^4^*F*_9/2_ spin-orbit level of the Nd^3+^ ions. Irrespective of the Nd^3+^ content, the room temperature emission spectra show radiative transitions from the ^4^*F*_3/2_ level into the ground levels ^4^*I*_9/2_ ($\langle \lambda_{\mathrm{em}}\rangle$ = 890 nm), ^4^*I*_11/2_ ($\langle \lambda_{\mathrm{em}}\rangle$ = 1063 nm) and ^4^*I*_13/2_ ($\langle \lambda_{\mathrm{em}}\rangle$ = 1341 nm). The ^4^*F*_3/2_ → ^4^*I*_11/2_ transition has the largest intensity, reflecting a large branching ratio of around *β*_11/2_(^4^*F*_3/2_) = 0.67 compared to the other observable radiative transitions (*β*_9/2_(^4^*F*_3/2_) = 0.17, *β*_13/2_(^4^*F*_3/2_) = 0.16). This is understandable as |ΔL| = 3, |ΔJ| = 4 implies a Judd-Ofelt allowed forced electric dipole transition and moreover, Nd^3+^ is typically characterized by large Ω_4_ and Ω_6_ Judd-Ofelt intensity parameters in phosphates [68,69]. Similar findings have been reported in the previously mentioned higher condensed phosphates ALa_1-*y*_Nd*_y_*(PO_3_)_4_ (A = Li – Rb; 0 ≤ *y* ≤ 1) [66]. As expected, the emission spectra become inhomogeneously broadened and the spectral resolution of the different crystal field components decreases with increasing Nd^3+^ content. The corresponding photoluminescence excitation spectra at room temperature acquired upon monitoring the most intense ^4^*F*_3/2_ → ^4^*I*_11/2_ emissive transition of Nd^3+^ at 1057 nm are depicted in Figure 4b). While the Kubelka-Munk spectra clearly suggest that the ^4^*I*_9/2_ → ^4^*F*_5/2_ transition at $\langle \lambda_{\mathrm{abs}}\rangle$ = 808 nm shows the maximum absorption coefficient, as expected for a Judd-Ofelt-allowed transition (|ΔL| = 3, |ΔJ| = 2), the excitation spectra reveal that absorption into any of the ^4^*F_J_* (*J* = 3/2…9/2) levels efficiently induces emission from the ^4^*F*_3/2_ level at room temperature, in particular with increasing Nd^3+^ content. Like in the emission spectra, the respective excitation transitions also suffer inhomogeneous broadening. Moreover, several artefacts due to the Xe lamp lines are present in the excitation spectra (see peaks marked with asterisks in Figure 4b). These artefacts can serve as an internal intensity standard. The strong decrease in relative intensity of Nd^3+^ excitation lines relative to the Xe-lamp lines with *x* indicates the presence of concentration quenching of the photoluminescence in the nanocrystals.

**Figure 5:**
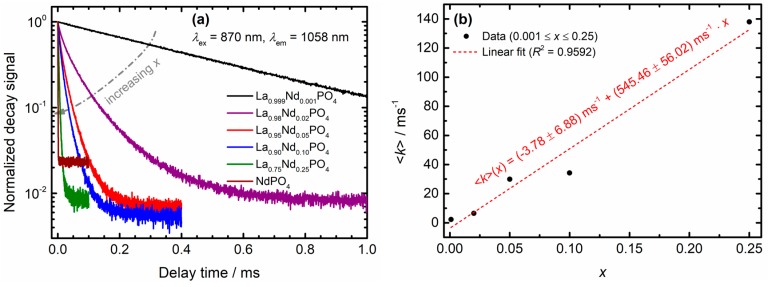

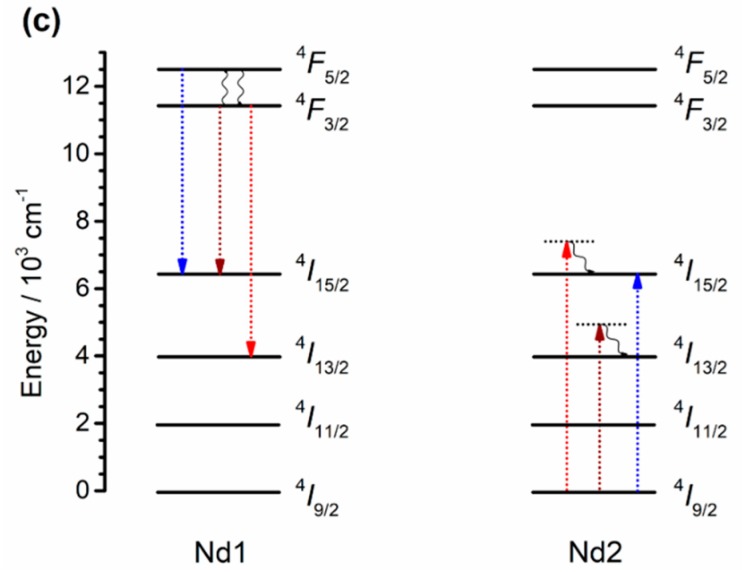
(**a**) Luminescence decay curves upon monitoring the ^4^*F*_3/2_ → ^4^*I*_11/2_ transition of the La_1−*x*_Nd*_x_*PO_4_ nanocrystals and the microcrystalline La_0.999_Nd_0.001_PO_4_ control sample at room temperature using pulsed direct excitation into the ^4^*F*_3/2_ level. The decay curve of a microcrystalline dilute La_0.999_Nd_0.001_PO_4_ powder is also depicted for comparison. (**b**) Plot of the average decay rates 〈k〉 obtained for the La_1−*x*_Nd*_x_*PO_4_ nanocrystals versus Nd^3+^ content *x*. The NdPO_4_ sample is excluded from the analysis. The red dashed line indicates a least-squares linear fit to the data. The intercept does not differ from zero at the statistical significance level of *α* = 0.05. (**c**) Schematic overview over the possible (eventually phonon-assisted) cross-relaxation processes that Nd^3+^ can undergo at higher concentrations. Blue arrows refer to cross-relaxation of the ^4^*F*_5/2_ level, brown and red arrows to cross-relaxation of the ^4^*F*_3/2_ level and curly arrows indicate emission or absorption of a resonant phonon.

Important information on radiative and non-radiative decay rates can be obtained from luminescence decay measurements. Figure 5a depicts the luminescence decay curves obtained upon direct excitation into the ^4^*F*_3/2_ level of Nd^3+^ at 870 nm and detection of the most intense ^4^*F*_3/2_ → ^4^*I*_11/2_ emissive transition around 1060 nm. The intensity of the ^4^*F*_3/2_–excited emission at *λ*_em_ = 1058 nm in a very dilute, microcrystalline La_0.999_Nd_0.001_PO_4_ control sample (*λ*_ex_ = 870 nm) decays monoexponentially with an (assumed purely radiative) decay rate of *k*_r_(^4^*F*_3/2_) = 2.25 ms^−1^ at room temperature (see Figure 5a). With increasing Nd^3+^ content, a faster and non-exponential decay is observed. This behavior is a clear signature of an energy transfer processes involving quenching of the ^4^*F*_3/2_ emission. In order to gain insight in the underlying quenching mechanism, the average decay rates 〈k〉 were determined with Equation (2) from the decay data depicted in Figure 5a.

(2) $$\langle k\rangle =\frac{1}{\langle \tau \rangle }=\frac{\sum_{j}I(t_{j})}{\sum_{j}t_{j}\;\times \;I(t_{j})}$$

In Equation (2), *I*(*t_j_*) denotes the normalized, background-corrected luminescence intensity at time *t_j_* and *j* runs over all acquired data points. In the case of NdPO_4_, the luminescence decay is already so fast that it was not feasible to reliably determine an average decay rate (see Figure 5a). Thus, it was excluded from further analysis. A plot of the average decay rates in La_1−*x*_Nd*_x_*PO_4_ versus the Nd^3+^ concentration *x* reveals an approximately linear relation between the two quantities (see Figure 5b). This is a clear signature of a two-ion process [70] and indicates that cross-relaxation of the ^4^*F*_3/2_ level between the Nd^3+^ ions becomes active in the LaPO_4_ host even at concentrations as low as 2 mol%. An explanation for the high cross-relaxation efficiency is the small nearest neighbor distance of Nd^3+^ ions in the orthophosphates (only 4.036 Å (NdPO_4_), 4.104 Å (LaPO_4_)) [60,61], which allows for an efficient electric dipole—electric dipole-type energy transfer. At lower concentrations and homogeneous doping, the average distances between Nd^3+^ ions will be larger and thus, the cross-relaxation efficiency is reduced. However, for electric dipole-electric dipole type energy transfer, cross-relaxation can be still effective over distances as large as 10 Å.

Inspection of the energy level diagram of Nd^3+^ shows that indeed the phonon-assisted cross-relaxation pathways [Nd1, Nd2]: [^4^*F*_3/2_, ^4^*I*_9/2_] → [^4^*I*_15/2_, ^4^*I*_13/2_] + $\hbar \omega_{\mathrm{eff}}$ and [^4^*F*_3/2_, ^4^*I*_9/2_] → [^4^*I*_13/2_, ^4^*I*_15/2_] + $\hbar \omega_{\mathrm{eff}}$ can take place if $\hbar \omega_{\mathrm{eff}}\approx$ 1050–1100 cm^−1^ (see Figure 5c). The required phonon energy agrees very well with the asymmetric O-P-O stretching vibration [59,66], whose energy also matches the energy gap between the ^4^*F*_5/2_ and ^4^*F*_3/2_ level. The ^4^*F*_5/2_ level is even prone to resonant cross-relaxation, [Nd1, Nd2]: [^4^*F*_5/2_, ^4^*I*_9/2_] → [^4^*I*_15/2_, ^4^*I*_15/2_] (see Figure 5c). The possibility of both the ^4^*F*_3/2_ and ^4^*F*_5/2_ level to decay via cross-relaxation effectively increases the decay rate of both spin-orbit levels. This is expected to have immediate consequences for the thermometric performance of Nd^3+^ once the average decay rates of those levels become similar to the non-radiative transition rates governing the thermal coupling of the excited states. If the average decay rates supersede the non-radiative transition rates to bridge the ^4^*F*_5/2_ – ^4^*F*_3/2_ gap, thermodynamic Boltzmann equilibrium cannot be sustained anymore, and the thermometric performance is lost.

**Figure 6:**
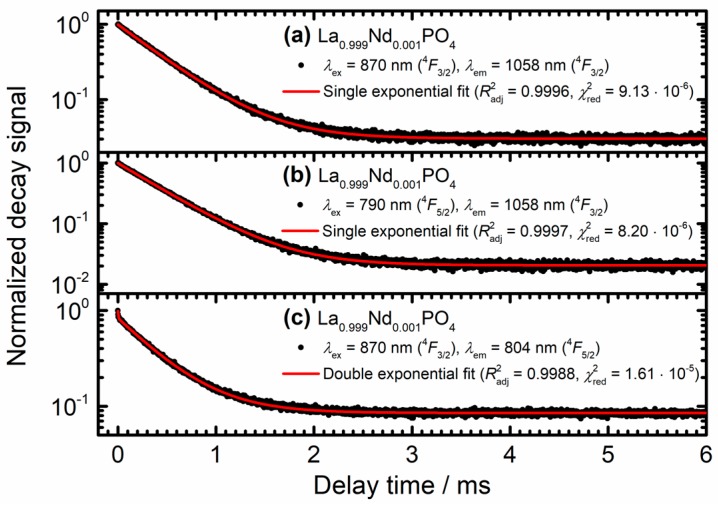
Luminescence decay curves of the Nd^3+^-related luminescence in microcrystalline La_0.999_Nd_0.001_PO_4_ powder. The respective excitation and emission wavelengths are given. Solid red lines depict least-squares fitting curves. The relevant statistical parameters indicating the quality of the fits are also indicated.

In order to obtain a semi-quantitative measure for the non-radiative transition rates mediating the thermal coupling between the ^4^*F*_5/2_ and ^4^*F*_3/2_ levels of Nd^3+^, luminescence decay curves upon selective excitation into each of those levels were recorded for the dilute microcrystalline La_0.999_Nd_0.001_PO_4_ sample. For excitation into either the ^4^*F*_3/2_ (*λ*_ex_ = 870 nm) or ^4^*F*_5/2_ level (*λ*_ex_ = 790 nm) and monitoring the luminescence decay of the ^4^*F*_3/2_ → ^4^*I*_11/2_-related transition at 1058 nm at room temperature, a purely single exponential decay with a radiative decay rate of *k*_r_(^4^*F*_3/2_) = 2.25 ms^−1^ is observed with errors well below 0.01 ms^−1^ (see Figure 6a,b).

**Figure 7:**
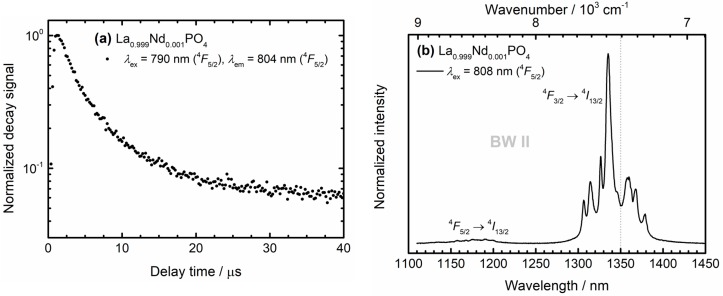
(**a**) Detailed view on the fast decay component from the ^4^*F*_5/2_ spin-orbit level of Nd^3+^ in microcrystalline La_0.999_Nd_0.001_PO_4_ powder. (**b**) High resolution emission spectrum depicting transitions from both the ^4^*F*_5/2_ and ^4^*F*_3/2_ level into the same ^4^*I*_13/2_ ground level acquired upon selected excitation into the ^4^*F*_5/2_ level (*λ*_ex_ = 808 nm) to separate the contribution of the non-radiative emission from the radiative emission on the decay rate of the ^4^*F*_5/2_ level.

This observation indicates that the sum of radiative and non-radiative decay rate from the ^4^*F*_5/2_ level, *k*_r_(^4^*F*_5/2_) + $k_{\mathrm{nr}}^{\mathrm{em}}\left(T\right)$, have to be much higher than the radiative decay rate *k*_r_(^4^*F*_3/2_) since otherwise a rise component is expected in the decay curve recorded for ^4^*F*_5/2_ excitation. For selective excitation at 870 nm (resonant with excitation into the ^4^*F*_3/2_ level) very weak anti-Stokes ^4^*F*_5/2_ emission is observed with a much faster initial decay component besides the slower decay of the overlapping ^4^*F*_3/2_ → ^4^*I*_9/2_ – related luminescence. This fast ~4 μs initial decay is assigned to the total decay rate of the ^4^*F*_5/2_ level. In order to obtain a more precise value, upon selective excitation into the ^4^*F*_5/2_ level, the luminescence decay was recorded for the strongest ^4^*F*_5/2_ → ^4^*I*_9/2_ – related emission at 804 nm (see Figure 7a). A fast decay with an average (radiative and non-radiative) decay rate of *k*_r_(^4^*F*_5/2_) + $k_{\mathrm{nr}}^{\mathrm{em}}\left(T=298\;K\right)$ = 204 ms^−1^ was found using Equation (2). The value agrees well with the result for the fast component from the double exponential fit (*k*_r_(^4^*F*_5/2_) + $k_{\mathrm{nr}}^{\mathrm{em}}\left(298\;K\right)$ = (242 ± 12) ms^−1^) as depicted in Figure 6c. For the following analysis, we will employ the average value *k*_r_(^4^*F*_5/2_) + $k_{\mathrm{nr}}^{\mathrm{em}}\left(298\;K\right)$ = (223 ± 19) ms^−1^.

In order to separate the contribution of radiative decay rate *k_r_*(^4^*F*_5/2_) from the non-radiative rate $k_{\mathrm{nr}}^{\mathrm{em}}\left(T\right)$ at temperature *T*, an emission spectrum containing the emissive transitions from both the ^4^*F*_5/2_ and ^4^*F*_3/2_ spin-orbit levels into the same ground level ^4^*I*_13/2_ was recorded for selective excitation into the ^4^*F*_5/2_ level (*λ*_ex_ = 808 nm). The ratio between the integrated intensities (if measured in photon counts) of the emission, *I*_em_, from the ^4^*F*_5/2_ and the ^4^*F*_3/2_ is equal to the ratio between *k*_r_(^4^*F*_5/2_) and $k_{\mathrm{nr}}^{\mathrm{em}}\left(T\right)$, respectively:

(3) kr(F45/2)knrem(T) =Iem(F45/2)Iem(F43/2)=β(F43/2→I413/2)Iem(F45/2→I413/2)β(F45/2→I413/2)Iem(F43/2→I413/2)

with β(F43/2→I413/2) = 0.16 and β(F45/2→I413/2) = 0.60 as the branching ratios of the respective transitions derived from the luminescence spectra (see Figure 4a and Figure 7b). Equation (3) is only valid if thermally excited non-radiative absorption from the ^4^*F*_3/2_ level back to the ^4^*F*_5/2_ is negligible and if there are no other non-radiative decay paths for the two levels.

The temperature dependence of non-radiative rates among 4*f^n^*-related spin-orbit levels is governed by thermally excited multi-phonon transitions. The thermal average number $\langle n_{\mathrm{eff}}\rangle$ of effective phonon modes with energy $\hbar \omega_{\mathrm{eff}}$ resonantly bridging the regarded energy gap between two electronic levels is given by the Planck formula:

(4) $$\langle n_{\mathrm{eff}}\rangle =\frac{1}{\exp\left(\frac{\hbar \omega_{\mathrm{eff}}}{k_{B}T}\right)-1}$$

with *k*_B_ as the Boltzmann constant. With an effective phonon energy of $\hbar \omega_{\mathrm{eff}}\approx$ 1050 cm^−1^, even at room temperature (*T* = 298 K), it is $\langle n_{\mathrm{eff}}\rangle \approx$ 6.34 ∙ 10^−3^, i.e., effectively, one phonon mode is only thermally excited with a very low probability. Based on Equation (3) and the emission spectrum depicted in Figure 7b, a non-radiative emission rate of $k_{\mathrm{nr}}^{\mathrm{em}}$(298 K) = *g*_1_*k*_nr_(0)$\left(1+\langle n_{\mathrm{eff}}\rangle \right)$ ≈ *g*_1_*k*_nr_(0) = (219 ± 19) ms^−1^ can be derived, where *g*_1_ = 4 is the (2*J* + 1)-fold degeneracy of the ^4^*F*_3/2_ level. Thus, the intrinsic non-radiative rate is estimated to be *k*_nr_(0) = (54.6 ± 4.7) ms^−1^.

The validity of the employed approximation of a still negligible thermal multi-phonon absorption rate for the non-radiative transition ^4^*F*_3/2_ → ^4^*F*_5/2_ can now be verified. With the value for *k*_nr_(0) and the degeneracy of the ^4^*F*_5/2_ level, *g*_2_ = 6, it is $k_{\mathrm{nr}}^{\mathrm{abs}}$(298 K) = *g*_2_*k*_nr_(0)$\langle n_{\mathrm{eff}}\rangle$ = (2.08 ± 0.18) ms^−1^, which agrees with *k*_r_(^4^*F*_3/2_) within the statistical error. Thus, the approximation to neglect that non-radiative absorption pathway for Nd^3+^ ions in the dilute La_0.999_Nd_0.001_PO_4_ microcrystals is just about to fail at room temperature and Boltzmann behavior should be expected to set in just above room temperature. In the case of the concentrated Nd^3+^-doped nanocrystals La_1−*x*_Nd*_x_*PO_4_, however, the additional cross-relaxation pathways and possible quenching of the ^4^*F*_3/2_ and ^4^*F*_5/2_ emission by high energy vibrations of any capping ligands such as -OH groups on the nanocrystal surface effectively increases the decay rate of the ^4^*F*_3/2_ and ^4^*F*_5/2_ level (see Figure 5a). Consequently, Boltzmann equilibrium is then expected to become active only at successively higher temperatures, once $k_{\mathrm{nr}}^{\mathrm{abs}}\left(T\right)$ supersedes these higher average decay rates. Table 2 compiles the relevant decay rates derived from decay dynamics and emission spectra of the dilute microcrystalline La_0.999_Nd_0.001_PO_4_ sample.

### 3.4. Consequences of Cross-Relaxation on Luminescence Thermometry Employing the ^4^F_5/2_ and ^4^F_3/2_ Spin-Orbit Levels of Nd^3+^

**Figure 8:**
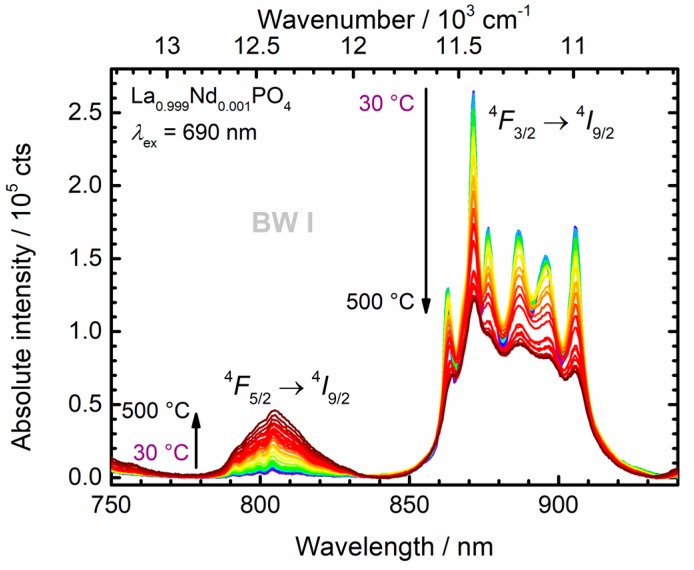
Exemplary temperature-dependent emission spectrum of La_0.999_Nd_0.001_PO_4_ in the spectral range of the first biological window (BW I). The thermally coupled radiative transitions are indicated.

For Boltzmann-based luminescence thermometry, it is beneficial to select a different auxiliary excited state that feeds the thermally coupled states giving rise to the temperature-dependent luminescence phenomena. Direct excitation into one of those levels provides an additional non-equilibrium component to the excited state population and although steady-state conditions might be retained, the single ion luminescence thermometer is driven out of thermodynamic equilibrium. Thus, despite the high absorption strength of the ^4^*I*_9/2_ → ^4^*F*_5/2_ transition at 808 nm, we decided to selectively excite into the ^4^*F*_9/2_ spin-orbit level with a corresponding absorption wavelength of 690 nm for all luminescence thermometry experiments. The temperature-dependent luminescence spectra of the microcrystalline La_0.999_Nd_0.001_PO_4_ upon excitation at 690 nm are depicted in Figure 8.

**Figure 9:**
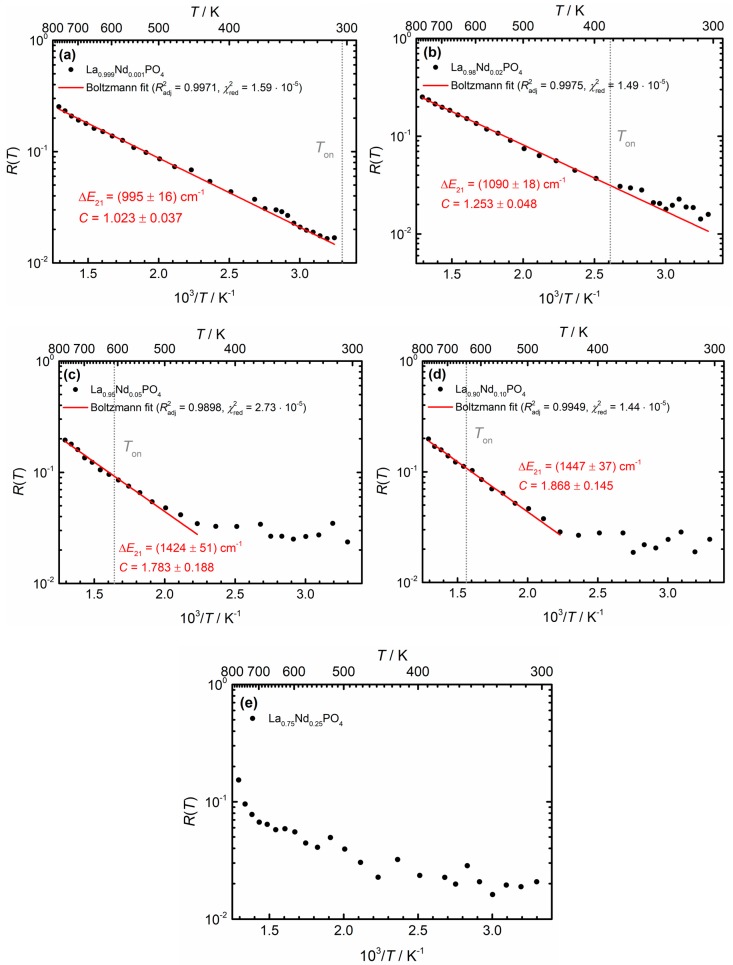
Semi-log plots of the temperature-dependent luminescence intensity ratio (LIR) between the ^4^*F*_5/2_ → ^4^*I*_9/2_- and ^4^*F*_3/2_ → ^4^*I*_9/2_-related emission of Nd^3+^ in (**a**) microcrystalline La_0.999_Nd_0.001_PO_4_, (**b**) nanocrystalline La_0.98_Nd_0.02_PO_4_, (**c**) La_0.95_Nd_0.05_PO_4_, (**d**) La_0.90_Nd_0.10_PO_4_ and (**e**) La_0.75_Nd_0.25_PO_4_. The NdPO_4_ nanocrystals were excluded from the analysis due to dominance of blackbody background already above 100 °C. Least-squares fits to the Boltzmann calibration law (see Equation (5)) are indicated by red solid lines together with the relevant statistical figures of merit. The predicted onset temperatures for Boltzmann equilibrium based on the decay analysis in Section 3.3 (see Figure 5a and Table 2) are also indicated.

All samples were investigated over a wide temperature range, also above physiological temperatures, for the sake of better insight into the thermal coupling between the ^4^*F*_3/2_ and ^4^*F*_5/2_ levels and to investigate the predicted thermometric performance at higher temperatures. In the case of thermodynamic equilibrium conditions between the two excited levels, the LIR, *R*(*T*), should obey Boltzmann’s law:

(5) $$R\left(T\right)=\frac{I_{20}}{I_{10}}=C\frac{g_{2}}{g_{1}}\exp\left(-\frac{{\Delta E}_{21}}{k_{B}T}\right),$$

where we denote the excited ^4^*F*_3/2_ level as state |1〉 and the ^4^*F*_5/2_ level as state |2〉 in the following. The ground level ^4^*I*_9/2_ is referred to as state |0〉. Then, *g*_1_ = 4 and *g*_2_ = 6 represent the (2*J* + 1)-fold degeneracies of the two spin-orbit levels, while Δ*E*_21_ = 1020 cm^−1^ as derived from the Kubelka-Munk spectra is the energy gap between the two excited levels. This value is in good agreement with expectations according to the Dieke—Carnall diagram [71]. *k*_B_ is Boltzmann’s constant and *C* is a pre-exponential factor that basically relates the electronic line strengths of the regarded radiative transitions of interest. It can also be estimated from Judd-Ofelt theory [14,69], but will be considered as a fitting parameter in the present study. Figure 9 depicts the thermometric Boltzmann plots derived from the temperature-dependent luminescence spectra of the La_1−*x*_Nd*_x_*PO_4_ nanocrystals in the wavelength range between 775 nm and 950 nm (i.e., in BW I). All spectra were corrected for constant instrumental background of the NIR PMT and additional blackbody radiation background, *B*(*λ*, *T*), at a given temperature *T* to obtain meaningful LIR data,

(6) $$B\left(\lambda ,\;T\right)=A+\frac{{2Dhc}^{2}}{\lambda^{5}\left[\exp\left(\frac{hc}{{\lambda k}_{B}T}\right)\;-\;1\right]}$$

with *A* and *D* as free parameters, *λ* as the wavelength, *h* as Planck’s constant and *c* as the light velocity. Due to concentration quenching the luminescence in NdPO_4_ was so weak, especially at temperatures above 100 °C, that no meaningful information could be extracted and thus, it was excluded from further analysis. The predictions according to the luminescence decay kinetics of Nd^3+^ can be directly compared to the experimental data. The LIR of the ^4^*F_J_* → ^4^*I*_9/2_ (*J* = 3/2, 5/2) transitions in La_0.999_Nd_0.001_PO_4_ shows Boltzmann behavior over the full temperature range from 30 °C to 500 °C. This observation is consistent with the observation that the non-radiative absorption rate $k_{\mathrm{nr}}^{\mathrm{abs}}$(*T*) governing the non-radiative ^4^*F*_3/2_ → ^4^*F*_5/2_ transition is similar to the radiative decay rate of the ^4^*F*_3/2_ level at room temperature and increases at higher temperatures. This will result in Boltzmann equilibrium starting just above room temperature. Overall, thermodynamic equilibrium between the two spin-orbit levels can be sustained over the full temperature range above ~300 K.

In the La_1−*x*_Nd*_x_*PO_4_ (*x* > 0.001) nanocrystals, the average decay rate of the ^4^*F*_3/2_ level increases with Nd^3+^ content due to the additional cross-relaxation pathways between neighboring Nd^3+^ ions. This additional decay channel competes with the non-radiative absorption rate and can hamper Boltzmann equilibration. Given the known temperature dependence of $k_{\mathrm{nr}}^{\mathrm{abs}}$(*T*) (scaling with the Planck factor in Equation (4)) and the faster decay of the ^4^*F*_3/2_ level (from the decay curves of the ^4^*F*_3/2_ emission in Figure 5), it is possible to determine the threshold temperature *T*_on_, above which Boltzmann behavior is expected in the more concentrated Nd^3+^-samples. *T*_on_ is taken as the temperature at which the non-radiative absorption $k_{\mathrm{nr}}^{\mathrm{abs}}$ becomes faster than the total decay rate of the ^4^*F*_3/2_ level. In very good agreement with expectations, a shifted onset of the Boltzmann behavior is observed in all higher concentrated La_1−*x*_Nd*_x_*PO_4_ nanocrystals, both experimentally and theoretically. The predicted onset temperatures *T*_on_ derived from the requirement of equal non-radiative absorption and average decay rates from the ^4^*F*_3/2_ level increases with Nd^3+^ content *x* and are indicated in Figure 9. For *x* = 0.02, Boltzmann equilibrium becomes problematic in the physiological temperature window and for *x* = 0.05, temperature sensing becomes possible only above 450 K. For the higher Nd^3+^ concentrations (*x* = 0.25) no Boltzmann behavior is observed even up to 500 °C. The predicted temperature at which the non-radiative absorption rate dominates is even higher, above 900 °C. Thus, although a higher Nd^3+^ content may increase the absorption efficiency of the nanocrystals (cf. Figure 3b), the efficient cross-relaxation of Nd^3+^ ions prevents sustainment of a Boltzmann equilibrium for the excited ^4^*F*_3/2_ and ^4^*F*_5/2_ levels for a larger temperature range. As a result, higher Nd^3+^ contents destroy the promising potential of the large ^4^*F*_5/2_–^4^*F*_3/2_ gap for Boltzmann thermometry with high sensitivity at physiological temperatures. In contrast, higher temperature thermometry (100 °C–500 °C) is still feasible even for Nd^3+^ concentrations as high as *x* = 0.10. Measurements of higher temperatures in BW I (and consequently, also in BW II) with those nanocrystals is however cumbersome since the blackbody background starts to dominate the emission spectrum and temperature can be measured more accurately from the background itself.

The effect of cross-relaxation on the useable temperature window for LIR temperature sensing can differ among lanthanide ions. Here we show for the ^4^*F*_3/2_ and ^4^*F*_5/2_ levels of Nd^3+^ that high dopant concentrations are clearly detrimental because cross-relaxation shortens the lifetime of the emitting levels, thus limiting the time available for Boltzmann equilibration. However, cross-relaxation can also be beneficial, if it provides an additional pathway for thermalization between the emitting levels, thus establishing Boltzmann equilibrium. This has been shown to be the case for the ^5^*D*_0_ and ^5^*D*_1_ levels of Eu^3+^ where cross-relaxation between neighboring Eu^3+^ ions provides an alternative path for relaxation and thus sustains Boltzmann behavior over a wider temperature range at elevated Eu^3+^ concentrations, as was demonstrated in the case of *β*-NaYF_4_:Eu^3+^ [58].

It is noteworthy that the fitted effective energy gaps, Δ*E*_21_, gradually increase with higher Nd^3+^ content *x*. This can be explained by the fact that the fitted effective energy gap for the nanocrystals with higher Nd^3+^ contents are obtained from higher temperature data. At higher temperatures, the probability for non-radiative absorption into the higher crystal field states of the ^4^*F*_5/2_ spin-orbit level increases, which effectively increases the energy gap Δ*E*_21_. Thus, only in the very dilute La_0.999_Nd_0.001_PO_4_ compound does the energy gap agree with the spectroscopically deduced energy gap according to the Kubelka-Munk spectra (Δ*E*_21_ = 1020 cm^−1^, see Figure 3a). Interestingly, also the pre-exponential constant *C* systematically increases with increasing Nd^3+^ content *x*. As it is fundamentally related to both the branching ratios and the radiative decay rates from the two considered excited states of Nd^3+^, its increase may also be related to the temperature-induced population of higher energetic crystal field states of the ^4^*F*_3/2_ and ^4^*F*_5/2_ levels. In conjunction with those observations, the predicted onset temperatures for Boltzmann behavior for the nanocrystals activated with 5 mol% and 10 mol% Nd^3+^ are significantly higher than the experimentally observed onsets (see Figure 9c,d). A possible explanation is a decreasing cross-relaxation efficiency of the ^4^*F*_3/2_ level at higher temperatures. Since cross-relaxation of the ^4^*F*_3/2_ level requires one high energy phonon mode of the phosphate host, an increasing temperature and population of the higher energy crystal field level may lead to an energy mismatch of the necessary energy transfer resonance condition and could thus reduce the cross-relaxation efficiency. Temperature-dependent luminescence decay analyses and modelling of the energy migration processes are necessary to confirm this hypothesis.

Since thermodynamic equilibrium between the ^4^*F*_5/2_ and ^4^*F*_3/2_ level of Nd^3+^ is sustained over the full temperature range investigated (30 °C–500 °C) in La_0.999_Nd_0.001_PO_4_, physiological temperatures are measurable by means of luminescence thermometry with that compound. As Boltzmann behavior is realized, the relative sensitivity *S*_r_ (in % K^−1^) of the luminescence thermometer is given by

(7) $$S_{r}=\left|\frac{1}{R(T)}\frac{dR}{dT}\right|=\frac{{\Delta E}_{21}}{k_{B}T^{2}}$$

**Figure 10:**
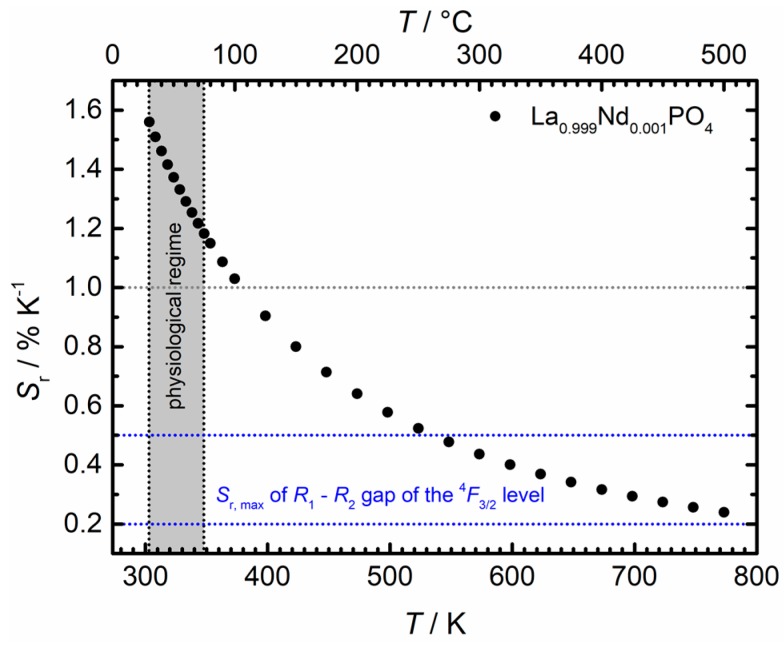
Temperature evolution of the relative sensitivity of Nd^3+^ in La_0.999_Nd_0.001_PO_4_ within the Boltzmann validity regime and upon usage of the ^4^*F*_5/2_–^4^*F*_3/2_ gap for Boltzmann-based luminescence thermometry. The grey dashed line indicates the desirable minimum relative sensitivity for in vivo luminescence thermometry, while the blue dashed regime marks the reported maximum achievable relative sensitivity for thermal coupling between the Kramers’ doublets of the ^4^*F*_3/2_ spin-orbit level [49,50,51].

Figure 10 depicts the evolution of the relative sensitivity for La_0.999_Nd_0.001_PO_4_ as obtained from Equation (7). In particular, it is higher than 1% K^−1^ for the full physiological temperature regime (30 °C–75 °C), which is practically difficult to achieve around room temperature with any single ion Boltzmann thermometer, especially in the NIR regime [49,50,51,57]. Typically, energy transfer-based thermometers are used in those cases [30,43,54,55] for which the underlying thermometric mechanisms are often not well established. The present results show promising potential of Nd^3+^ for physiological temperature sensing by means of luminescence thermometry, if the boundary conditions for the validity of a Boltzmann equilibrium are met which requires low Nd^3+^ concentrations. The ^4^*F*_5/2_–^4^*F*_3/2_ spin-orbit gap in Nd^3+^ gives rise to relative sensitivities that are an order of magnitude higher than the typically found ones in the range of 0.25% K^−1^ [50]. A disadvantage of the presented thermometric concept of Nd^3+^ is the rather low intensity of the ^4^*F*_5/2_ → ^4^*I*_9/2_ emission that can give rise to a higher relative intensity uncertainty, depending on the sensitivity of the detection system. Since it is the temperature uncertainty that matters in a well performing luminescence thermometer, both the relative sensitivity and emission intensities have to be optimized.

## 4. Conclusions

Nd^3+^ is a promising candidate for in vivo luminescence thermometry due to its intense radiative transitions in the biological transparency windows BW I and BW II. Most Nd-based single ion thermometers utilize the two crystal field states arising from the excited ^4^*F*_3/2_ spin-orbit level. These thermometers are fundamentally limited in their relative thermal sensitivity *S*_r_ due to the small energy gap of ~100 cm^−1^ and the high demands on spectral resolution which are difficult to meet under in vivo conditions. An alternative promising probe for physiological temperature sensing is the ^4^*F*_5/2_–^4^*F*_3/2_ spin-orbit gap of Nd^3+^ which is ~1000 cm^−1^. In order to investigate the feasibility of temperature sensing based on the temperature dependent emission intensity ratio of ^4^*F*_3/2_- and ^4^*F*_5/2_-related emission, both microcrystalline, dilute La_0.999_Nd_0.001_PO_4_ and nanocrystalline La_1−*x*_Nd*_x_*PO_4_ (*x* = 0.02, 0.05, 0.10, 0.25, 1.00) were synthesized by means of a co-precipitation approach and structurally, morphologically and optically characterized. Analysis of the decay kinetics reveals that the ^4^*F*_3/2_ and ^4^*F*_5/2_ excited states of Nd^3+^ are prone to efficient cross-relaxation quenching at higher Nd-concentrations. Cross-relaxation competes with the non-radiative transition rates governing the thermalization of the ^4^*F*_5/2_ and ^4^*F*_3/2_ spin-orbit levels. This additional decay pathway leads to a gradual breakdown of Boltzmann equilibrium at physiological temperatures with increasing Nd^3+^ concentration. Only for low Nd^3+^ concentrations (*x* < 0.02) is it possible to sustain Boltzmann equilibrium between the ^4^*F*_5/2_ and ^4^*F*_3/2_ levels of Nd^3+^ in the physiological temperature regime. The relative sensitivity in this important temperature regime exceeds 1% K^−1^. Overall, the present study demonstrates that a careful analysis of the excited state dynamics allows for an assessment of the performance of a luminescence thermometer, but also demonstrates the potential pitfalls of Boltzmann thermometry that can be encountered. A mechanistic understanding of the competition between radiative and non-radiative decay processes can lead to a tailored design of novel luminescence thermometers with optimal performance in a specific temperature window.

## Figures and Tables

**Table 1:** Average crystallite size estimates 〈d〉, lattice parameters *a*, *b*, *c*, cell volume *V* and strain of nanocrystals according to Rietveld refinement on the XRPD patterns depicted in Figure 1. The numbers in brackets refer to the errors in the last digits, respectively. The quality parameters for the Rietveld refinement are given below: Profile factor *R*_p_, weighted profile factor *R*_wp_ and optimum expected *R*_exp_ factor together with the final goodness of the fit (G.o.f.) to the structural input of the monazite structure type.

	La_0.98_Nd_0.02_PO_4_	La_0.95_Nd_0.05_PO_4_	La_0.90_Nd_0.10_PO_4_	La_0.75_Nd_0.25_PO_4_	NdPO_4_
〈d〉/nm	9.239(17)	6.852(18)	6.652(6)	9.057(19)	6.199(18)
*a*/Å	6.8631(13)	6.8504(14)	6.8592(19)	6.8390(15)	6.8380(4)
*b*/Å	7.1043(13)	7.0901(13)	7.0924(19)	7.0799(15)	6.9890(4)
*c*/Å	6.5290(12)	6.5171(12)	6.5141(18)	6.5053(14)	6.4210(4)
*V*/Å^3^	309.84(21)	308.08(39)	308.44(23)	306.57(26)	298.67(11)
Strain %	0.37(2)	0.39(5)	0.34(2)	0.50(8)	0.42(6)
*R*_p_/%	5.79	6.22	6.58	5.93	7.80
*R*_wp_/%	4.36	4.61	4.85	4.47	5.78
*R*_exp_/%	3.84	3.94	3.81	3.68	3.13
G.o.f.	1.50	1.58	1.72	1.61	2.49

**Table 2:** Derived radiative and non-radiative rates characterizing the thermal coupling of the ^4^*F*_3/2_ and ^4^*F*_5/2_ spin-orbit levels of Nd^3+^ in microcrystalline La_0.999_Nd_0.001_PO_4_.

*k*_r_(^4^*F*_3/2_)/ms^−^^1^	*k*_r_(^4^*F*_5/2_)/ms^−1^	*k*_nr_(0)/ms^−1^	$k_{\mathrm{nr}}^{\mathrm{em}}$(298 K)/ms^−1^	$k_{\mathrm{nr}}^{\mathrm{abs}}$(298 K)/ms^−1^
2.25	3.16 ± 0.27	54.6 ± 4.7	219 ± 19	2.08 ± 0.18
